# Impact of Trauma Team Census on Patients With Rib Fractures

**DOI:** 10.7759/cureus.84479

**Published:** 2025-05-20

**Authors:** Anthony J Duncan, David R Velez, Khaled Zreik

**Affiliations:** 1 Department of Surgery, University of North Dakota School of Medicine and Health Sciences, Grand Forks, USA; 2 Department of Surgical Critical Care, Sanford Medical Center, Fargo, USA

**Keywords:** acute delirium, intensive care unit (icu), resident workload, rib fractures, trauma centers

## Abstract

Background: Workloads of healthcare professionals have continued to increase in recent years. With increased workload, some studies have shown increased length of stay for patients, higher cost, and higher risk of patient safety events. Trauma teams are a group that is particularly vulnerable to changes in patient census. This study aims to evaluate the impact of high versus low trauma team census on outcomes.

Methods: A retrospective review of all rib fracture patients at a level I trauma center from 2017 to 2022 was conducted. The average daily census was determined, and high or low census days were assessed by being above or below this, respectively. The impact of census levels on outcomes was assessed using bivariate analysis and multivariate regression.

Results: A total of 1,291 patients were included, with 663 admitted on high census days and 628 admitted on low census days. Demographics and comorbidities were similar between groups. Surgical fixation rates and time to surgery were similar between census levels. High census admissions had lower ICU admission rates (odds ratio (OR): 0.78, p=0.034) and higher delirium risk (OR: 3.21, p=0.005).

Conclusion: Despite some seasonal variation between groups, patients admitted on high and low census days have similar demographics and prehospital comorbidities. However, patients admitted on high census days were less likely to receive ICU admission and had increased delirium rates, raising concerns about under-triage and emphasizing the need for improved resource allocation during high-volume periods.

## Introduction

Physician workloads have steadily increased with the evolving landscape of healthcare delivery [1]. Recent studies have begun to evaluate the negative effect of physician and team census on patient outcomes [2,3]. Patient census has been associated with a higher admission rate in the emergency room [4]. Within hospitalist groups, increased workload has been associated with increased length of stay, higher costs, lower teaching effectiveness, and higher risk of patient safety events [2,3]. Nearly 25% of hospitalists admit to ordering potentially unnecessary tests, procedures, or consults due to insufficient time for in-person patient evaluations [5]. The data is controversial, however, as other studies demonstrate only minor differences in resource use with no significant difference in patient outcomes [6].

Higher ICU occupancy days have been associated with higher rates of premature ICU discharge within the ICU, but data on mortality differences are conflicting [7-9]. Increased hospital occupancy has been significantly linked to delays in ICU transfers [10]. In the pediatric ICU, rounding teams have disproportionately shortened the time spent discussing the plan of care for low-acuity patients on high census days. However, this difference was not seen in high-acuity patients [11].

A prior work by our group looking at outcomes in trauma patients based on team census found increased rates of delirium and decreased rates of ICU admission in traumatic brain injury (TBI) patients [12]. We anticipate similar findings in rib fractures. This study aims to evaluate the impact of trauma team census in patients with traumatic rib fractures.

## Materials and methods

Study setting

Our facility is a level 1 trauma center in the Upper Midwest. The Trauma and Acute Care Surgery (TRACS) team manages trauma and emergency general surgery patients. The team is staffed by a rotating faculty of seven surgeons fellowship-trained in surgical critical care. The resident team typically consists of a chief resident and 2-3 junior residents, working with 1-3 nurse practitioners. Provider staffing is not adjusted based on patient census, resulting in significant variability in the patient-to-provider ratio, ranging from one provider for every two patients on low census days to one provider for as many as 14 patients during peak times.

Data collection

We conducted a five-year retrospective review of all trauma patients admitted to our center between July 1, 2017, and June 30, 2022. Daily patient census was determined by the number of patients with an active Trauma and Acute Care Surgery order in the electronic medical record system. The mean census across the five years was used to define high and low census.

Demographic information was collected, including age, gender, and race. Comorbidities for patients were collected, which included alcohol abuse, angina, anticoagulation, bleeding disorders, chemotherapy, cirrhosis, congestive heart failure, chronic obstructive pulmonary disease (COPD), dementia, diabetes, hemodialysis, hypertension, myocardial infarction (MI), peripheral arterial disease, pregnancy, smoker, steroid use, stroke, and substance use. Mechanism of injury was obtained for both groups and compared. We compared the census to the vital signs upon arrival (heart rate, respiratory rate, pulse oximetry, systolic blood pressure, and temperature), Glasgow Coma Scale (GCS) score, Injury Severity Score (ISS), and concomitant injuries. Lastly, complications, hospital length of stay, ICU length of stay and ventilation days, and discharge disposition were compared for patients admitted on low census days to those admitted on high census days.

Statistical analysis

Our statistical analysis was conducted with the utmost fairness and objectivity. Parametric variables were evaluated using Student's T-test. Qualitative and non-parametric variables were assessed using the Mann-Whitney U test. The Shapiro-Wilk test was used to confirm a non-normal distribution for hospital length of stay, ICU length of stay, and ventilator days with a significant right skew. Multivariate linear and logistic regression was performed for complications and outcomes, adjusting for the number of rib fractures. Statistical analyses were performed using Stata version 16.0 (StataCorp LLC, College Station, TX). Tests of significance were two-sided, with p-values considered significant at the 0.05 level.

## Results

A total of 4,696 trauma patients were seen between July 1, 2017, and June 30, 2022. A total of 1,291 (27.5%) patients sustained rib fractures. The average patient census was 41.5, ranging from 17 to 70. The average patient census was used to determine high and low census groups, with days being above or below, respectively. Of patients with traumatic rib fractures, 51% (n=663) were admitted on days with a low (below average) census, and 49% (n=628) were admitted on days with a high (above average) census. From May to October, the average patient census was 44.4, and from November to April, it was 36.8 (p<0.001). Patient demographics and comorbidities for patients admitted on high census days were compared to those admitted on low census days. Patients admitted on high census days had lower rates of hypertension. There were no other significant differences (Table 1).

**Table 1:** Demographics and comorbidities ^1^Mean, T-test ^2^% (number), Chi-square *p<0.05 COPD: chronic obstructive pulmonary disease

Variables	Low census (n=663)	High census (n=628)	p-value
Demographics			
Age (years)^1^	57.4	56.1	0.283
Male sex^2^	67.6% (448)	66.6% (418)	0.699
White^2^	86.0% (570)	85.5% (537)	0.812
Black^2^	1.5% (10)	1.4% (9)	0.911
Native American^2^	10.1% (67)	10.8% (68)	0.672
Asian^2^	1.1% (7)	0.8% (5)	0.628
Comorbidities			
Alcohol abuse^2^	11.6% (77)	10.0% (63)	0.361
Angina^2^	1.8% (12)	3.2% (20)	0.113
Anticoagulation^2^	13.7% (91)	12.9% (81)	0.662
Bleeding disorder^2^	1.7% (11)	3.2% (20)	0.074
Chemotherapy^2^	1.2% (8)	1.6% (10)	0.555
Cirrhosis^2^	2.1% (14)	1.0% (6)	0.093
Congestive heart failure^2^	6.0% (40)	7.2% (45)	0.413
COPD^2^	7.8% (52)	6.4% (40)	0.304
Dementia^2^	3.2% (21)	1.9% (12)	0.153
Diabetes^2^	16.7% (111)	14.0% (88)	0.175
Hemodialysis^2^	0.8% (5)	0.8% (5)	0.932
Hypertension^2^	40.9% (271)	35.0% (220)	0.028*
Myocardial Infarction^2^	1.1% (7)	0.3% (2)	0.112
Peripheral arterial disease^2^	0.6% (4)	1.3% (8)	0.210
Pregnant^2^	0.0% (0)	0.2% (1)	0.305
Smoker^2^	33.6% (223)	34.1% (214)	0.837
Steroid use^2^	2.6% (17)	2.9% (18)	0.739
Stroke^2^	1.7% (11)	2.1% (13)	0.585
Substance abuse^2^	8.7% (58)	6.5% (41)	0.134

The mechanism of injury is compared in Table 2. Patients admitted on high census days were considerably more likely to have suffered a motorcycle crash or all-terrain vehicle (ATV) crash and significantly less likely to have suffered a snowmobile crash.

**Table 2:** Mechanism of injury ^1^% (number), Chi-square *p<0.05 ATV: all-terrain vehicle

Variables	Low census (n=663)	High census (n=628)	p-value
Fall^1^	43.0% (285)	39.8% (250)	0.247
Motor vehicle crash^1^	28.1% (186)	30.3% (190)	0.385
Motorcycle crash^1^	5.3% (35)	8.9% (24)	0.011*
ATV crash^1^	4.1% (27)	7.0% (19)	0.021*
Snowmobile crash^1^	3.2% (21)	0.2% (1)	<0.001*
Bicycle crash^1^	1.4% (9)	2.2% (14)	0.237
Pedestrian trauma^1^	2.6% (17)	2.4% (15)	0.840
Sport trauma^1^	0.3% (2)	0.0% (0)	0.169
Watersport trauma^1^	0.2% (1)	0.2% (1)	0.970
Machining trauma^1^	0.8% (5)	0.6% (4)	0.801
Animal trauma^1^	0.5% (3)	0.5% (3)	0.947
Assault^1^	2.4% (16)	1.4% (9)	0.202
Gunshot wound^1^	1.7% (11)	1.1% (7)	0.405
Stab wound^1^	0.5% (3)	0.5% (3)	0.947

Presentation, rib fracture description, and concomitant injuries were compared in Table 3. Patients admitted on high census days had significantly higher temperatures, but no other differences in presentation or concomitant injury were seen.

**Table 3:** Presentation and concurrent injuries ^1^Mean ^2^% (number), Chi-square *p<0.05 GCS: Glasgow Coma Scale, ISS: Injury Severity Score, TBI: Traumatic Brain Injury

Variables	Low census (n=663)	High census (n=628)	p-value
Presentation			
Heart rate (bpm)^1^	86.7	87.3	0.565
Respiratory rate (bpm)^1^	18.8	18.6	0.656
Pulse oximetry^1^	96%	95%	0.818
Systolic blood pressure (mmHg)^1^	133.4	132.6	0.792
Temperature (°C)^1^	36.6	36.8	0.004*
GCS^1^	13.6	13.6	0.584
ISS^1^	16.6	16.9	0.387
Rib fractures			
Single rib fracture^2^	18.2% (121)	14.5% (91)	0.068
Multiple rib fractures^2^	76.9% (506)	79.9% (502)	0.189
Flail segment^2^	6.2% (41)	7.0% (44)	0.552
Concomitant injuries			
TBI^2^	25.8% (171)	26.4% (166)	0.793
Skull fracture^2^	9.2% (61)	7.8% (49)	0.369
Facial fracture^2^	11.3% (75)	11.5% (72)	0.931
C-spine fracture^2^	12.4% (82)	12.9% (81)	0.775
T/L-spine fracture^2^	32.4% (215)	32.8% (206)	0.886
Spinal cord injury^2^	5.4% (36)	5.6% (35)	0.910
Spleen^2^	8.1% (54)	7.6% (48)	0.804
Liver^2^	7.4% (49)	7.0% (44)	0.907
Kidney^2^	3.6% (24)	2.9% (18)	0.257
Pancreas^2^	0.6% (4)	0.3% (2)	0.453
Adrenal^2^	2.7% (18)	2.2% (14)	0.575
Stomach^2^	0.5% (3)	0.2% (1)	0.344
Duodenum/small intestine^2^	1.7% (11)	1.3% (8)	0.692
Colon^2^	1.4% (9)	1.9% (12)	0.908
Rectum^2^	0.0% (0)	0.0% (0)	NA
Pulmonary^2^	45.9% (304)	49.4% (310)	0.207
Cardiac^2^	1.2% (8)	1.4% (9)	0.722
Thoracic vascular injury^2^	1.4% (9)	2.1% (13)	0.323
Abdominal vascular injury^2^	1.2% (8)	1.1% (7)	0.878
Clavicle fracture	10.3% (69)	10.8% (68)	0.738
Scapula fracture^2^	9.2% (61)	11.9% (75)	0.109
Pelvic fracture^2^	12.7% (84)	14.3% (90)	0.383

Of patients admitted on low census days, 5.1% (n=34) underwent surgical fixation of the ribs, compared to 4.9% (n=31) for patients admitted on high census days (p=0.875). On low census days, the average time to surgical fixation of the ribs was 4.3 days, compared to 4.1 days on high census days (p=0.509).

After adjusting for the number of rib fractures, 36% (n=239) of patients with rib fractures were admitted to the ICU on low census days, compared to 30.3% (n=190) on high census days (odds ratio (OR) (95% confidence interval): 0.78 (0.62-0.98), p=0.034). Complications and outcomes, adjusted for the number of rib fractures, were compared in Table 4 and Table 5, respectively. Patients with rib fractures admitted on high census days had a significantly higher risk of developing delirium (p=0.005). There were no other differences in complications or outcomes.

**Table 4:** Comparison of complications for patients with rib fractures admitted on low census days to those admitted on high census days, adjusted for the number of rib fractures *p<0.05

Variables	Low census (n=663)	High census (n=628)	Adjusted odds ratio (95% confidence interval)	p-value
Any complication	0.133	0.127	0.95 (0.69-1.31)	0.747
Acute kidney injury	0.017	0.011	0.66 (0.25-1.71)	0.393
Delirium	0.012	0.038	3.21 (1.43-7.21)	0.005*
Cardiopulmonary resuscitation	0.008	0.010	1.23 (0.37-4.06)	0.731
Deep venous thrombosis	0.026	0.025	0.99 (0.96-1.04)	0.975
Myocardial infarction	0.005	0.010	1.83 (0.44-7.73)	0.408
Pressure ulcer	0.021	0.014	0.77 (0.32-1.83)	0.549
Pulmonary embolism	0.006	0.014	2.32 (0.71-7.59)	0.163
Stroke	0.005	0.002	0.35 (0.04-3.41)	0.368
Urinary tract infection	0.009	0.006	0.70 (0.20-2.49)	0.580

**Table 5:** Comparison of outcomes for patients with rib fractures admitted on low census days to those admitted on high census days, adjusted for the number of rib fractures

Variables	Low census (n=663)	High census (n=628)	Coefficient (95% confidence interval)	p-value
Hospital length of stay (days)	8.0	7.6	-0.04 (-0.09-0.01)	0.121
ICU length of stay (days)	5.9	5.6	-0.02 (-0.08-0.06)	0.643
Ventilator days (days)	7.5	5.9	-0.06 (-0.16-0.04)	0.270
Variables	Low census (n=663)	High census (n=628)	Adjusted odds ratio (95% confidence interval)	p-value
Discharge disposition				
Home	0.508	0.505	1.00 (0.99-1.02)	0.585
Home health	0.063	0.062	1.00 (0.98-1.03)	0.975
Rehab	0.137	0.162	1.01 (0.99-1.03)	0.216
Nursing home	0.125	0.118	0.99 (0.97-1.01)	0.215
Swing bed	0.023	0.032	0.99 (0.96-1.04)	0.925
Long-term acute care	0.050	0.030	0.97 (0.94-1.01)	0.106
Death	0.050	0.041	0.99 (0.96-1.02)	0.528
Against medical advice	0.009	0.005	0.57 (0.14-2.28)	0.424

## Discussion

Patient census was higher from May to October compared to November to April, reflecting the significant seasonal variations in temperature and snowfall. During this colder period, the average low temperature in Fargo, ND, is approximately 14.3°F (-9.8°C) [13,14]. Mechanisms of injury appear to correlate with these changes, as high census days were more likely to have suffered a motorcycle crash or ATV crash but less likely to have sustained a snowmobile crash. Similarly, patients admitted on low census days had a lower average temperature. Patients admitted on high census days had lower rates of hypertension, but otherwise had no significant difference in age, demographics, or underlying comorbidities.

Aside from likely climate-related variations, the high and low census groups were largely comparable. There were no significant differences in presenting vital signs, GCS, ISS, or concomitant injuries. However, after adjusting for the number of fractures, patients with rib fractures admitted on high census days were 16% less likely to be admitted to the ICU, which raises concern that these patients could potentially be being under-triaged due to the higher workloads.

After controlling for the number of rib fractures, patients admitted on high census days showed no significant differences in overall morbidity or mortality. Rates of surgical rib fixation and time to surgery were also comparable. However, these patients faced a significantly higher risk of delirium, with over triple the odds ratio. Delirium is a severe and common complication seen in patients with rib fractures and is associated with longer hospital length of stay and higher risk of institutionalization [15]. Increased awareness is imperative in this population. Numerous interventions have been associated with prevention and improved outcomes [16,17]. A key concern is whether patients receive insufficient attention or care during periods of high census and increased workloads. There were no other differences in outcomes or discharge disposition. These findings mirror what we saw in our prior paper, looking at TBI patients based on census data, with higher rates of delirium and lower rates of ICU admission in patients who were admitted on high patient census days [12].

There are potential limitations to this review. First, the effects of team census on outcomes are likely context-dependent and may have different findings in other settings. Second, the lower-than-expected complication rates may indicate underreporting within our system, potentially confounding the results. Third, although we accounted for numerous patient variables, other facility factors such as changes in ancillary staff coverage and general hospital-specific changes could not be accounted for. These limitations underscore the need for further research in this area to better understand the impact of patient census on patient care outcomes and develop strategies to mitigate potential issues.

## Conclusions

As physician workloads continue to increase, the effects on patient outcomes must be monitored. In this analysis evaluating patients with traumatic rib fractures, patients admitted on high census days had no significant differences in outcomes, rates of surgical fixation, or time to surgery. Patients admitted on high census days were less likely to be admitted to the ICU, raising concerns for under-triage. These patients also had increased rates of delirium, possibly indicating that they were receiving less attention and care during their stay due to an increased workload. This data emphasizes the need to maintain quality care despite increasing workloads. Groups must plan for these changes and adapt to high-volume periods.
